# The molecular and cellular basis of olfactory response to tsetse fly attractants

**DOI:** 10.1371/journal.pgen.1008005

**Published:** 2019-03-15

**Authors:** J. Sebastian Chahda, Neeraj Soni, Jennifer S. Sun, Shimaa A. M. Ebrahim, Brian L. Weiss, John R. Carlson

**Affiliations:** 1 Dept. of Molecular Cellular and Developmental Biology, Yale University, New Haven, Connecticut, United States of America; 2 Dept. of Epidemiology of Microbial Diseases, Yale School of Public Health, New Haven, Connecticut, United States of America; National Centre for Biological Sciences, TIFR, INDIA

## Abstract

Dipteran or “true” flies occupy nearly every terrestrial habitat, and have evolved to feed upon a wide variety of sources including fruit, pollen, decomposing animal matter, and even vertebrate blood. Here we analyze the molecular, genetic and cellular basis of odor response in the tsetse fly *Glossina morsitans*, which feeds on the blood of humans and their livestock, and is a vector of deadly trypanosomes. The *G*. *morsitans* antenna contains specialized subtypes of sensilla, some of which line a sensory pit not found in the fruit fly *Drosophila*. We characterize distinct patterns of *G*. *morsitans Odor receptor (GmmOr)* gene expression in the antenna. We devise a new version of the “empty neuron” heterologous expression system, and use it to functionally express several GmmOrs in a mutant olfactory receptor neuron (ORN) of *Drosophila*. GmmOr35 responds to 1-hexen-3-ol, an odorant found in human emanations, and also alpha-pinene, a compound produced by malarial parasites. Another receptor, GmmOr9, which is expressed in the sensory pit, responds to acetone, 2-butanone and 2-propanol. We confirm by electrophysiological recording that neurons of the sensory pit respond to these odorants. Acetone and 2-butanone are strong attractants long used in the field to trap tsetse. We find that 2-propanol is also an attractant for both *G*. *morsitans* and the related species *G*. *fuscipes*, a major vector of African sleeping sickness. The results identify 2-propanol as a candidate for an environmentally friendly and practical tsetse attractant. Taken together, this work characterizes the olfactory system of a highly distinct kind of fly, and it provides an approach to identifying new agents for controlling the fly and the devastating diseases that it carries.

## Author summary

African trypanosomiasis, also known as “sleeping sickness” in humans and “wasting disease” in livestock, is a lethal and economically burdensome disease that afflicts rural communities throughout sub-Saharan Africa. The parasite that causes the disease is spread by the tsetse fly, which feeds on the blood of humans and their livestock. One effective strategy to control tsetse and the diseases that it carries has been to attract the fly into traps, or repel the fly, with odorants. The efficiency of this approach can be greatly improved through understanding of the molecular and cellular machinery of tsetse olfaction, and by identification of odorants that manipulate it. Here we provide findings on the anatomical, cellular, and molecular underpinnings of tsetse olfaction, which previously has been greatly understudied. We characterize the expression of odor receptors in the antenna and study their function with a new version of an *in vivo* expression system. We identify receptors that respond to ecologically relevant odors in the tsetse fly's environment. One receptor, which is expressed in an unusual pit structure, responds to odorants that attract tsetse and may mediate attraction. We identify an odorant that activates this receptor and that could be useful in combatting African trypanosomiasis.

## Introduction

Tsetse flies are of special interest in two respects. First, they are vectors of human and animal diseases that have had an enormous impact on health and economic development in major portions of sub-Saharan Africa. Second, they have a highly unusual and intriguing lifecycle (reviewed in [1]).

Tsetse feed exclusively on the blood of vertebrate animals. Upon feeding, the flies can transmit trypanosomes. In humans the trypanosomes may cause sleeping sickness, also known as human African trypanosomiasis; 70 million people are at risk of this disease [2]. In livestock the trypanosomes cause nagana, also called African animal trypanosomiasis. The combined losses to livestock and to the potential for agricultural development are estimated at $4 -$4.5 billion US annually [3].

Tsetse are biologically different from other flies in several ways. They are classified as a distinct family, the Glossinidae. Inside a female fly, a single egg is fertilized, hatches and then develops as a larva within the uterus. The larva is nourished internally by a milk-like fluid produced by the mother. The larva develops through two stages, and then during a third stage the larva emerges from the female fly, burrows into the ground, and pupariates. After several weeks an adult ecloses from the pupal case. The larva does not feed on external food sources, and thus the entire developmental program of the animal depends on energy from bloodmeals taken by the mother. To find human or animal sources of blood, tsetse flies rely largely on olfaction.

**Fig 1:**
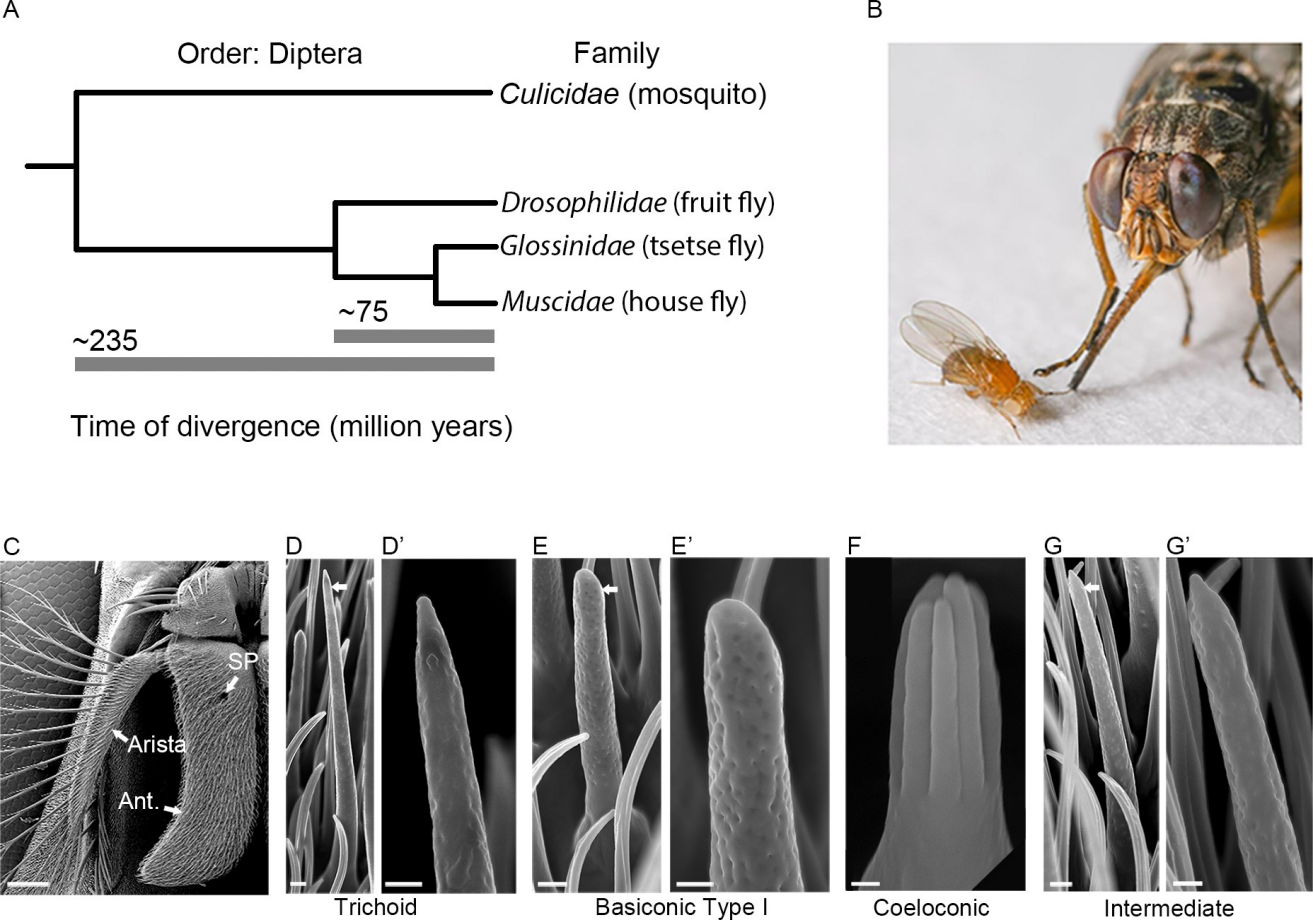
The relationship of *Glossina morsitans* to *Drosophila melanogaster* and its antennal sensilla. (A) Phylogenetic tree showing the evolutionary relationship among four families within the order Diptera: Culicidae, Drosophilidae, Glossinidae, and Muscidae. Estimated divergence times are from Wiegmann et al., 2011 [4]. (B) Photograph of *D*. *melanogaster* and *G*. *m*. *morsitans* courtesy of Dr. Geoffrey Attardo (adapted from Sun et al. 2018 [27]). (C) Scanning electron micrograph of the antenna of *G*. *morsitans*. Arrows indicate the third antennal segment (Ant.), arista, and sensory pit (SP). Micrographs of trichoid (D, D’), basiconic (E, E’), coeloconic (F), and intermediate (G, G’) olfactory sensilla from *G*. *morsitans*. Arrows in D, E, and G indicate the sensilla that are shown at higher magnifications in the images to the right. Scale bars = 100 μm for C; 1 μm for D, E, G; 0.5 μm for D’, E’, G’; and 0.25 μm for F.

Fly olfaction has been studied extensively in the fruit fly *Drosophila melanogaster*. However, *Drosophila* diverged from tsetse ~75 million years ago [4] (Fig 1A), is much smaller (Fig 1B), does not produce milk, and feeds on fermenting fruits rather than blood. Analysis of the tsetse olfactory system is of interest in part to understand how it compares to that of *Drosophila*. However, analysis of tsetse olfaction is also critical to understanding the mechanism by which it finds its human and animal hosts, which could in turn lead to new means of disease control.

One of the most effective ways of controlling human and animal trypanosomiasis has been to reduce the population of tsetse using traps baited with attractive odorants. Two odorants that have been found effective as baits in attracting tsetse are acetone and butanone, both of which are emanated by oxen and cows [5–9]. Artificial baits generally use either acetone or butanone, but not both; these two odorants are believed to be able to substitute for each other [5,6].

Here we analyze the olfactory system of the tsetse fly *Glossina morsitans*, a vector of both human and animal trypanosomiasis. We examine by electron microscopy its olfactory sensilla and two sensory invaginations of the antennal surface, one of which is shared with *Drosophila* and one of which is not. We characterize the spatial expression patterns of a subset of *G*. *morsitans* odor receptor genes *(GmmOrs)*. We then develop a new version of an *in vivo* functional expression system for Ors, and use it to analyze the response profiles of three tsetse odor receptors. We find that one receptor responds strongly to both acetone and 2-butanone, suggesting a molecular basis for long-standing conclusions drawn from control efforts in the field. We also identify another odorant that excites this receptor, that attracts tsetse, and is an appealing candidate for a practical control agent. Taken together, our results suggest a strategy for identifying odorants that may be useful in controlling tsetse and the devastating diseases that it spreads.

## Results

### Olfactory sensilla of the *G*. *morsitans* antennal surface

We began our analysis by examining the olfactory sensilla on the surface of the *G*. *morsitans* antenna via scanning electron microscopy (Fig 1C). The third antennal segment is more elongated than its *Drosophila* counterpart, but is similar in that it is covered with several morphological classes of sensilla [10].

Trichoid sensilla on the *G*. *morsitans* antenna are ~22–24 μm in length and taper to a fine tip (Fig 1D and 1D’). They are strikingly similar to those in *D*. *melanogaster*, which are 18–22 μm in length and detect pheromones [11–14].

Basiconic sensilla on the surface of the *G*. *morsitans* antenna are ~10–12 μm in length (Fig 1E and 1E’). Their rounded tips, their pores, and the morphology of their basal structures are also reminiscent of those in *D*. *melanogaster*. The surface of the *Drosophila* antenna contains three types of basiconic sensilla, ranging from 9–12 μm in height and classified as large, small, and thin [10], all of which detect food odors [15]. The basiconic sensilla on the surface of the *G*. *morsitans* antenna appear more uniform in size and are referred to here as “basiconic Type I”.

Grooved coeloconic sensilla, ~3 μm in length, were also observed at low density on the *G*. *morsitans* antennal surface (Fig 1F). These sensilla are comprised of conjoined, finger-like protrusions that form grooves. In *Drosophila*, there are coeloconic sensilla on the antennal surface that appear similar in structure [10] and that respond to amines and organic acids [16,17].

The *G*. *morsitans* antenna also contains sensilla that are intermediate in morphology between trichoid and basiconic sensilla (Fig 1G and 1G’). Similar intermediate sensilla have been described in *Drosophila* [10], where they detect food odors [18,19].

In *Drosophila*, each morphological class of sensillum is restricted to a region of the antennal surface; the regions occupied by each class overlap, but are distinct [10]. Although we have not mapped the sensillum types on the *G*. *morsitans* surface exhaustively, we have observed a greater degree of overlap than in *Drosophila*, *i*.*e*. most sensillum types can be found on most portions of the antennal surface.

### Invaginations of the *G*. *morsitans* antenna and their sensilla

**Fig 2:**
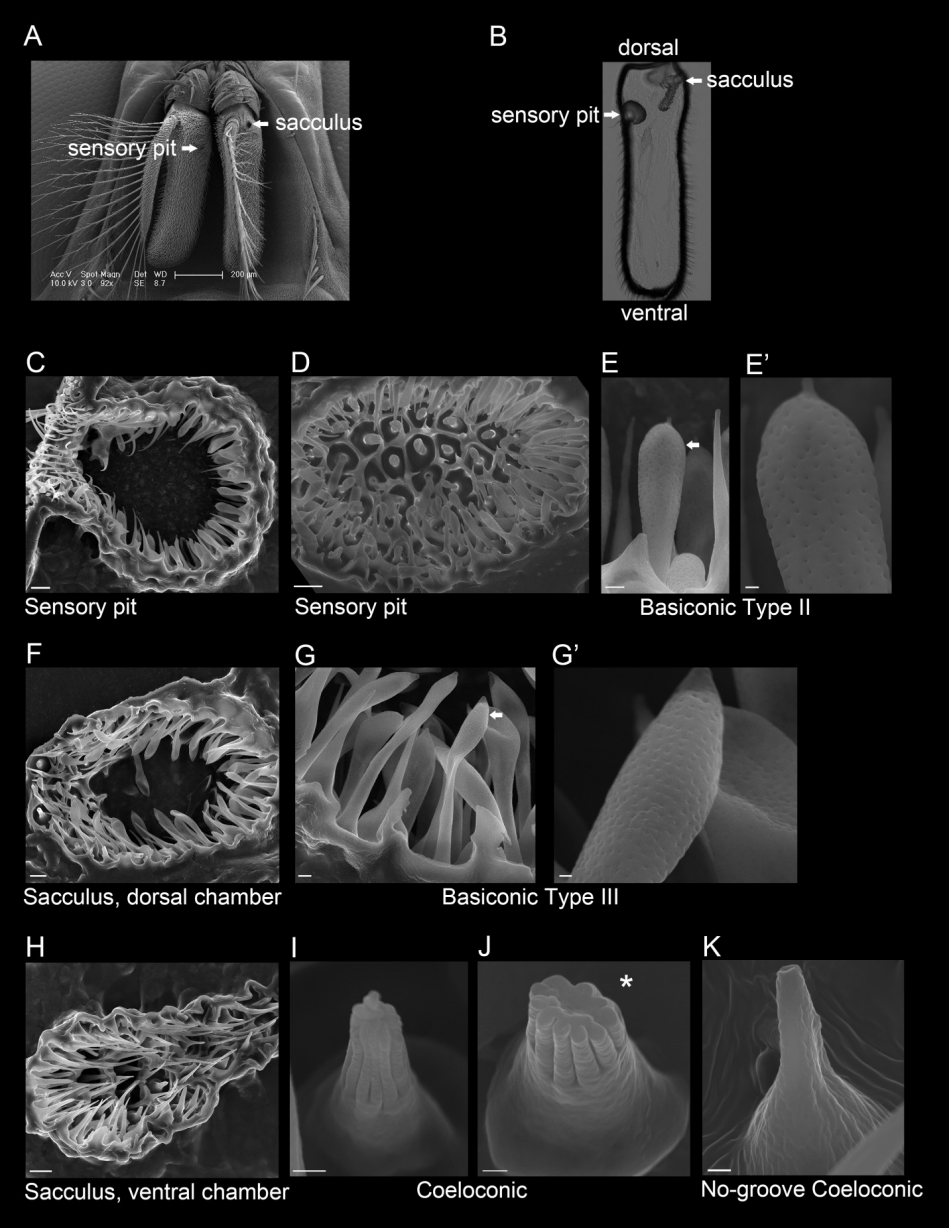
The sensory pit and sacculus of the *G*. *morsitans* antenna. (A) Scanning electron micrograph of a *G*. *morsitans* head with arrows indicating the openings of the sensory pit and sacculus. (B) Transmitted light image of an antennal cross section (coronal plane) showing the sensory pit and sacculus that open to the medial and lateral sides of the antenna, respectively. (C) Micrograph of a sectioned sensory pit with its opening to the antennal surface at the upper left. (D) Micrograph of the sensory pit, showing a top-down view of the olfactory sensilla that line the pit. (E, E’) The sensory pit is lined with basiconic Type II sensilla. The arrow in E indicates the sensillum shown in E’. (F) Dorsal chamber of the sacculus (medial is left, lateral is right) that is lined with basiconic Type III sensilla (G, G’). The arrow in G indicates the sensillum shown in G’. (H) Ventral chamber of the sacculus (medial is at left, lateral is at right) that is lined with coeloconic (I, J) and no-groove coeloconic sensilla (K). The asterisk in panel J marks an axial view of a coeloconic sensillum that had its tip cut off during cryosectioning, fortuitously revealing its inner structure. Scale bars = 200 μm for A; 5 μm for C, D, F and H; 1 μm for E, G; and 0.25 μm for E’, G’, I, J, and K.

There are two openings, one on each side of the *G*. *morsitans* antenna, that lead to invaginations, or cavities, within the antenna (Fig 2A). Tissue sectioning shows that the medial opening leads to a sensory pit and the lateral opening leads to a two-chambered sacculus (Fig 2B). In order to examine sensilla within the sensory pit and sacculus, antennae were cryosectioned and imaged by scanning electron microscopy.

The sensory pit is a single cavity that opens to the medial side of the antenna (Fig 2C and 2D). It contains basiconic sensilla that are distinct from the Type I basiconic sensilla observed on the antennal surface, in that they are club-like in shape and ~2 μm shorter, *i*.*e*. ~8–10 μm in length (Fig 2E and 2E’). We will refer to these sensilla as basiconic Type II sensilla. They are morphologically distinct from any sensilla on the *D*. *melanogaster* antenna.

The sacculus is divided into two chambers, one dorsal and one ventral, which both feed into the same opening on the lateral side of the antenna. The dorsal chamber (Fig 2F) is lined with basiconic sensilla that are similar to Type II but are ~13–15 μm in length (Fig 2G and 2G’); we refer to them as basiconic Type III sensilla. These Type III sensilla also have a distinct morphology from any on the antenna of *D*. *melanogaster*. The ventral chamber (Fig 2H) is lined with grooved coeloconic sensilla (Fig 2I and 2J) as well as coeloconic sensilla that do not contain grooves (Fig 2K), both of which are ~3 μm in length.

The *Drosophila* antenna also contains a sacculus, but it contains three chambers rather than two. The *Drosophila* sacculus contains grooved sensilla that detect acids [20], and ungrooved coeloconic sensilla that respond to humidity [21–23]. *Drosophila* does not contain a sensory pit, raising interesting questions about the function of this cavity in tsetse.

### Conservation of molecular identity of sensillum classes

As detailed above, the *G*. *morsitans* antenna contains sensilla that are morphologically similar to those on the *D*. *melanogaster* antenna, *e*.*g*. trichoid, basiconic, and coeloconic sensilla. Are they also similar in terms of their molecular identity? In *Drosophila*, different morphological classes of sensilla express different *Odorant binding protein* genes *(Obps)* in their underlying support cells [24]. For example, *Obp76a* is expressed in trichoid sensilla; *Obp28a* is expressed in basiconic sensilla; *Obp84a* and *Obp59a* are expressed in coeloconic sensilla. Each of these genes has a single ortholog within the family of ~30 *G*. *morsitans Obp* genes [25], so we asked whether each of these four *GmmObp* orthologs is expressed in the same morphological class of sensilla in tsetse as its counterpart in *Drosophila*.

**Fig 3:**
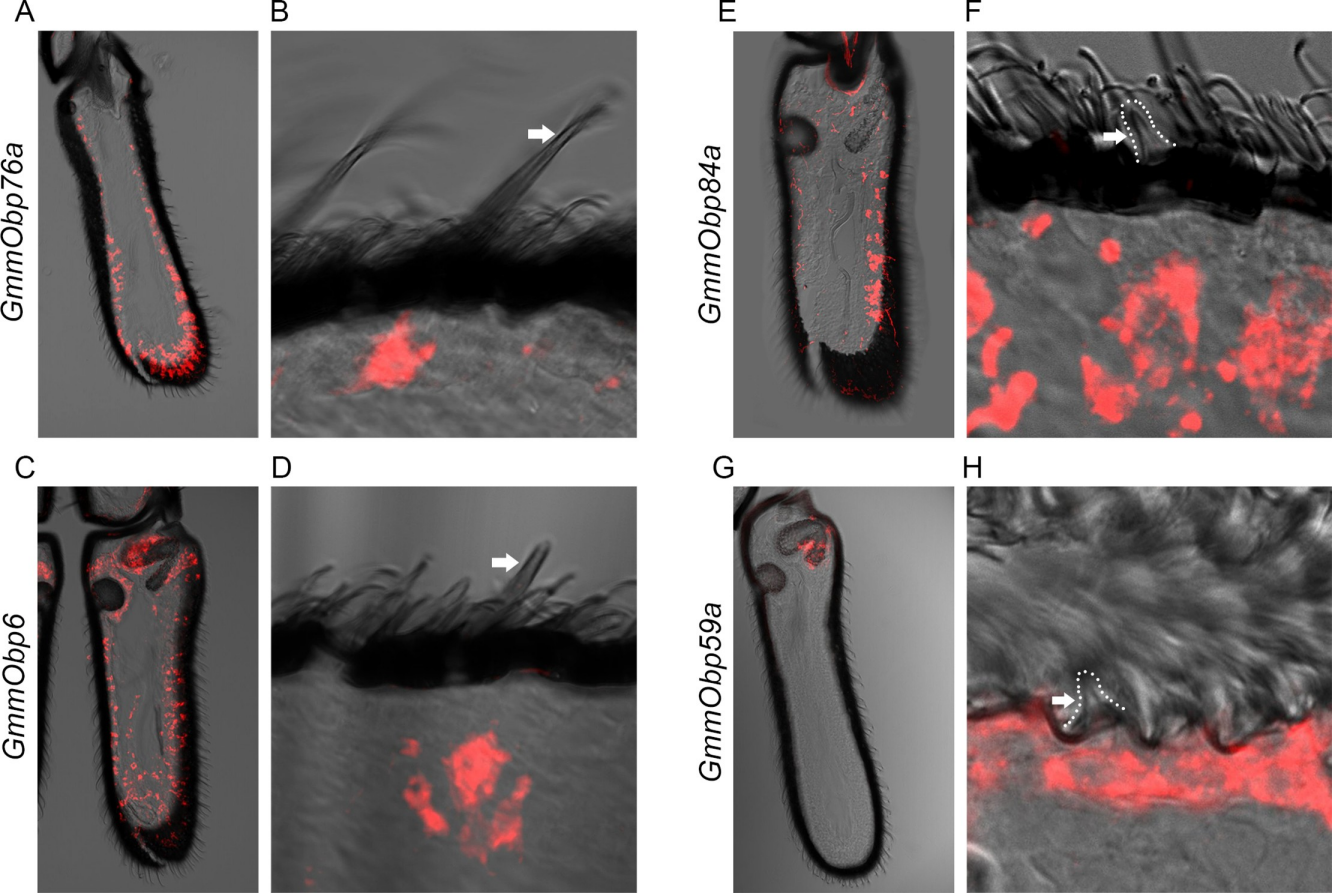
Expression of *Odorant binding protein* genes. (A, B) *In situ* hybridization to *GmmObp76a* in (A) 40 μm and (B) 10 μm antennal cross sections. Arrow in panel B marks a trichoid sensillum in the same plane as a cell body marked by *GmmObp76a*. (C, D) *GmmObp6* in (C) 40 μm and (D) 10 μm antennal cross sections. Arrow in D marks a basiconic sensillum in the same plane as a cell body marked by *GmmObp6*. (E, F) *GmmObp84a* in (E) 40 μm and (F) 10 μm antennal cross sections. Arrow in F marks a coeloconic sensillum in the ventral chamber of the sacculus in the same plane as a cell body marked by *GmmObp84a*. (G, H) *GmmObp59a* (red) in (G) 40 μm and (H) 10 μm antennal cross sections. Arrow in H marks a coeloconic sensillum in the same plane as a cell body marked by *GmmObp84a*.

*GmmObp76a*, the tsetse ortholog of *Obp76a*, also known as *Lush*, is detected by *in situ* hybridization broadly across the antenna (Fig 3A). When thinner antennal sections were analyzed (10 μm vs. 40 μm sections) to isolate a single layer of sensilla, *GmmObp76a* could be observed to label cells directly beneath the shaft of a trichoid sensillum (Fig 3B), consistent with its expression in *Drosophila*.

*GmmObp6* (also designated *GmmOBP6* [26]), the tsetse ortholog of *Obp28a*, was detected more evenly across the antenna, including the sensory pit and the dorsal chamber of the sacculus (Fig 3C), which agrees with the distribution of basiconic sensilla in tsetse. Moreover, analysis of thinner sections revealed labeling of basiconic sensilla (Fig 3D).

*GmmObp84a*, the tsetse ortholog of *Obp84a*, was detected in a pattern consistent with that of coeloconic sensilla found on the antennal surface (Fig 3E). Analysis of thinner sections revealed labeling associated with coeloconic sensilla (Fig 3F). Finally, *GmmObp59a*, the tsetse ortholog of *Obp59a*, has earlier been shown to map to the tsetse sacculus (Fig 3G, and [27]). More detailed analysis confirmed this labeling and revealed that Obp59a is expressed in a region that contains only a single class of sensory hairs, coeloconic sensilla, consistent with its expression in coeloconic sensilla in *Drosophila* (Fig 3H).

In summary, tsetse contains trichoid, basiconic, and coeloconic sensilla that are similar to their *Drosophila* counterparts not only in morphology but also in expression of at least some odorant binding proteins.

### Spatial organization of *Ors* on the antennal surface

**Fig 4:**
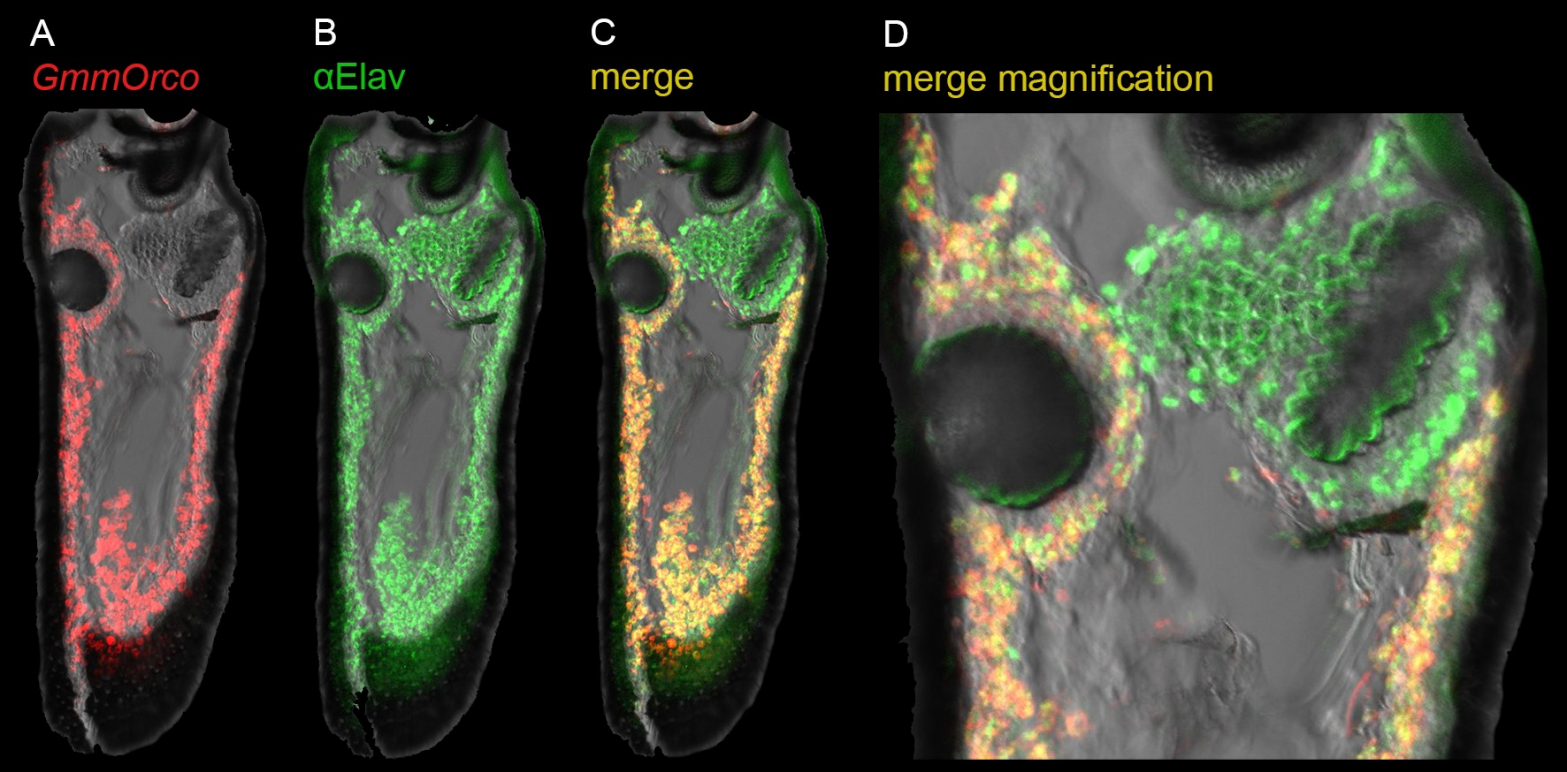
Expression of *GmmOrco* in the antenna. (A) *In situ* hybridization to *GmmOrco* (red). (B) Immunostaining of pan-neuronal marker Elav (green). (C) Merged image of *GmmOrco* and anti-E lav. (D) Magnification of (C).

**Fig 5:**
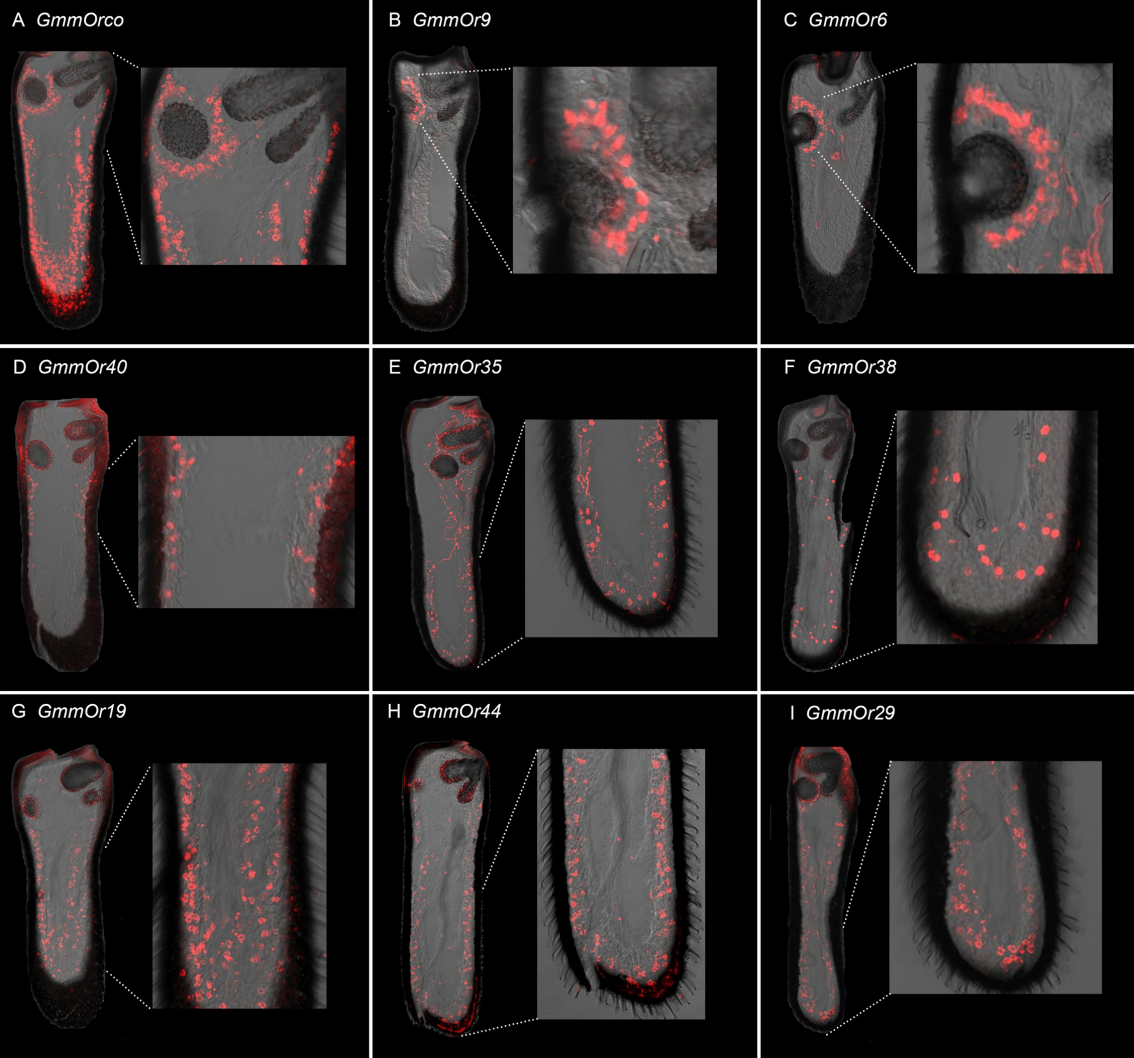
Expression of *GmmOrs* in the antenna. For all images dorsal is up and medial is to left. (A) *GmmOrco*. (B) *GmmOr9*. (C) *GmmOr6*. (D) *GmmOr40*. (E) *GmmOr35*. Some background fluorescence is visible that does not emanate from cell bodies; see sense strand control in S1H Fig. (F) *GmmOr38*. (G) *GmmOr19*. (H) *GmmOr44*. (I) *GmmOr29*. We note that in several of these images there is background fluorescence that emanates from the cuticle, which is thicker in the antenna of *G*. *morsitans* than in *D*. *melanogaster*. See S1 Fig for sense strand controls.

As a first step in examining the spatial organization of *Odorant receptor (Or)* expression in the tsetse antenna, we analyzed the expression of *GmmOrco*, the tsetse ortholog of *Orco*. In *Drosophila*, Orco is an essential co-receptor for Ors [28], and serves as a marker for Or-expressing olfactory receptor neurons (ORNs). *In situ* hybridization with a *GmmOrco* probe showed broad labeling across the tsetse antenna (Figs 4A and 5A; results with a sense strand control are shown in S1 Fig).

We then carried out a double-label experiment with the *GmmOrco* probe and an anti-Elav antibody, which is a pan-neuronal marker. When tested alone, the anti-Elav antibody labels cells in a broad distribution across the antenna (Fig 4B). When the anti-Elav antibody is used together with the *GmmOrco* probe, we observed co-labeling across most of the antenna (Fig 4C), with the exception of a region surrounding the sacculus where we predominantly observe Elav^+^ *GmmOrco*^*-*^ cells (Fig 4D). These results are consistent with those found in *Drosophila*, where most ORNs surrounding the sacculus do not express *Orco* but rather express members of the *Ionotropic Receptor (IR)* family [29].

The first bioinformatic search for *GmmOrs* identified ~46 genes [25]. We tested 29 of these *GmmOrs* by *in situ* hybridization with RNA probes, and identified eight that consistently produced relatively strong labeling in the antenna. These eight *GmmOrs* are diverse: some but not others have *Drosophila* orthologs, and the expression and function of the orthologs in *Drosophila* are diverse.

*GmmOr9* showed expression in cells surrounding the sensory pit (Fig 5B; a sense strand control is shown in S1 Fig). There was little if any specific labeling of cell bodies elsewhere. *GmmOr6* also showed strong labeling of cells around the sensory pit (Fig 5C). By contrast, other *GmmOrs* showed different labeling patterns. *GmmOr40* expression is localized to the midsection of the antenna (Fig 5D), whereas many of the cell bodies labeled by *GmmOr35* are located in the distal region of the antenna (Fig 5E). We note that a sense strand control for *GmmOr35* and some other probes show some background cuticular fluorescence that does not arise from cell bodies (S1 Fig; the cuticle of the *G*. *morsitans* antenna is thicker than that in *D*. *melanogaster*, and a wide variety of conditions and probe lengths were tried to minimize background fluorescence). *GmmOr38* also shows strong labeling in the distal region, but more proximal labeling is clear as well (Fig 5F). *GmmOr19*, *GmmOr44* and *GmmOr29* label cell bodies located along the entire antennal axis (Fig 5G, 5H and 5I). All eight *GmmOrs* showed similar expression in male and female antennae.

### Two *Ors* are expressed in neighboring neurons of the sensory pit

We were interested to find that *GmmOr6* and *GmmOr9* both showed labeling near the sensory pit. To examine the relationship between their expression patterns at higher resolution, we carried out a series of double-label experiments.

**Fig 6:**
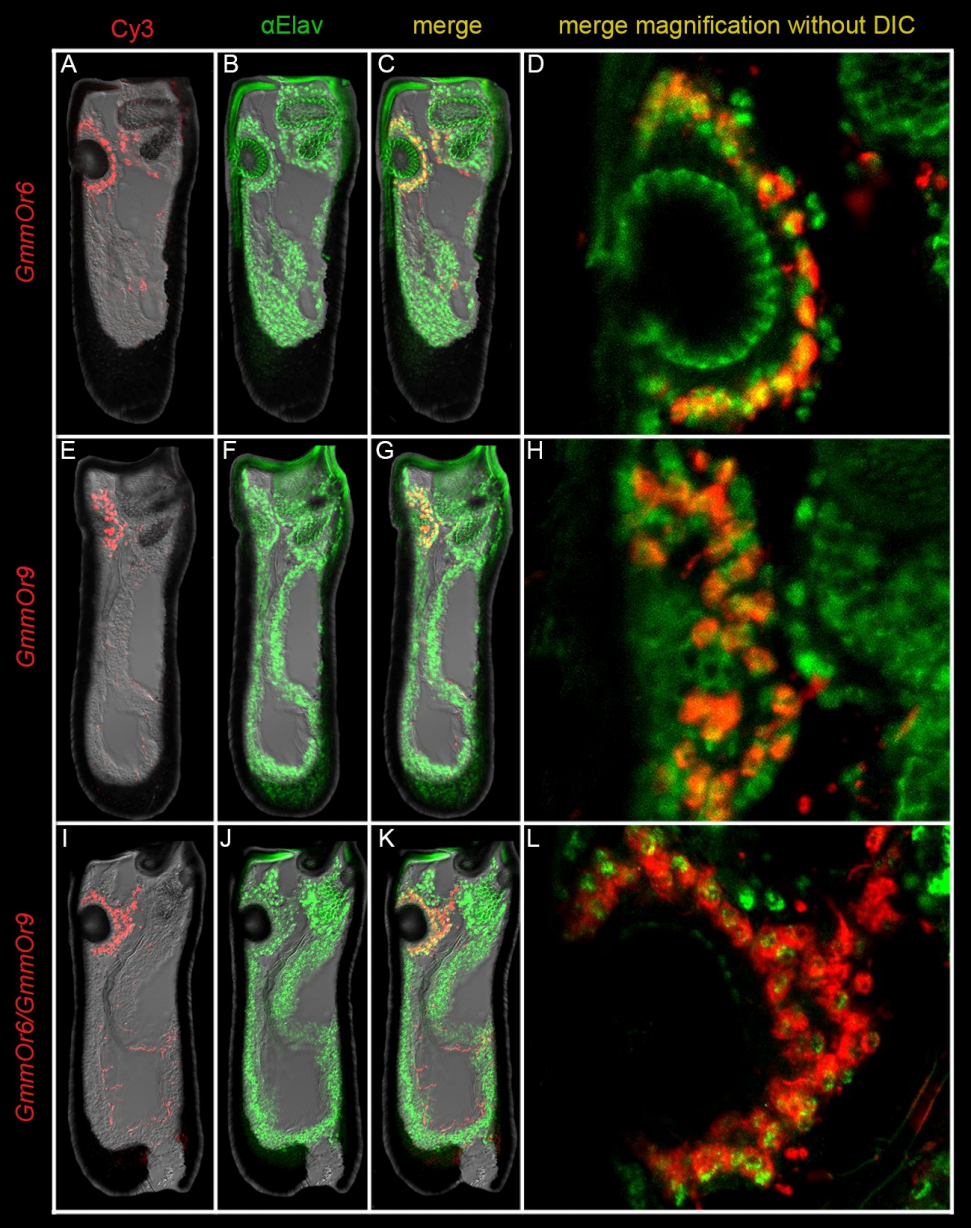
*GmmOr6* and *GmmOr9* are transcribed in adjacent ORNs that encapsulate the sensory pit. (A-D) *In situ* hybridization to *GmmOr6* (red) combined with anti-Elav immunostaining (green). (A) *GmmOr6*, (B) anti-Elav, and (C) merged image of the whole antenna. (D) Magnified merged image of the sensory pit without DIC. The circular structure in the center of the image that is uniformly green is autofluorescent cuticle of the pit. Likewise, the green fluorescence in the upper right corner is cuticular autofluorescence from the sacculus. (E-H) *In situ* hybridization to *GmmOr9* (red) combined with anti-Elav immunostaining (green). (E) *GmmOr9*, (F) anti-Elav, and (G) merged image of the whole antenna. (H) Magnified merged image of the sensory pit without DIC. (I-L) Double *in situ* hybridization to *GmmOr6* and *GmmOr9* (both red) combined with anti-Elav immunostaining (green). (I) *GmmOr6* and *GmmOr9*, (J) anti-Elav, and (K) merged image of the whole antenna. (L) Magnified merged image of the sensory pit without DIC.

First, we performed a double-label experiment with an *in situ* hybridization probe against *GmmOr6*, which labels ORNs around the sensory pit, and the anti-Elav antibody, which is expected to label all ORNs (Fig 6A–6D). The results showed that *GmmOr6* labels about half of the ORNs near the sensory pit (Fig 6D). Most *GmmOr6*^+^ ORNs appear in close proximity to a *GmmOr6*^***-***^ ORN.

We then carried out a double-labeling experiment with *GmmOr9* and the anti-Elav antibody (Fig 6E–6H). *GmmOr9* also labeled about half of the ORNs around the sensory pit, and most individual *GmmOr9*^*+*^ ORNs appeared close to a *GmmOr9*^***-***^ ORN (Fig 6H). Finally, we carried out a triple-labeling experiment with the *GmmOr6* and *GmmOr9* probes, and the anti-Elav antibody (Fig 6I–6L). In this case, all or almost all Elav^+^ ORNs around the sensory pit were labeled with a *GmmOr* probe (Fig 6L). The simplest interpretation of these results is that there are two kinds of ORNs that innervate the sensory pit, one expressing *GmmOr6* and one expressing *GmmOr9*, and that they are paired within the same sensillum type.

### Creation of a new “empty neuron” system with CRISPR-Cas9

The “empty neuron” system has been used to analyze the functional properties of a wide variety of odor receptors from *Drosophila* [15], the mosquitoes *Anopheles gambiae* and *Aedes aegypti* [30,31], and other insects, including Lepidopterans [32]. The system is based on a mutant antennal neuron of *Drosophila*, the ab3A neuron, that lacks a functional odor receptor [33]. Exogenous receptors are expressed in ab3A with the *GAL4-UAS* system, and odorant responses are then measured electrophysiologically from this neuron.

The empty neuron system in its original form has used (i) a deletion that removes the adjacent *Or22a* and *Or22b* genes, (ii) an *Or22a-GAL4* construct, and (iii) a *UAS-OrX* construct. The deletion suffers impaired viability in the homozygous state, presumably because of the loss of sequences flanking the *Or22a/b* locus.

**Fig 7:**
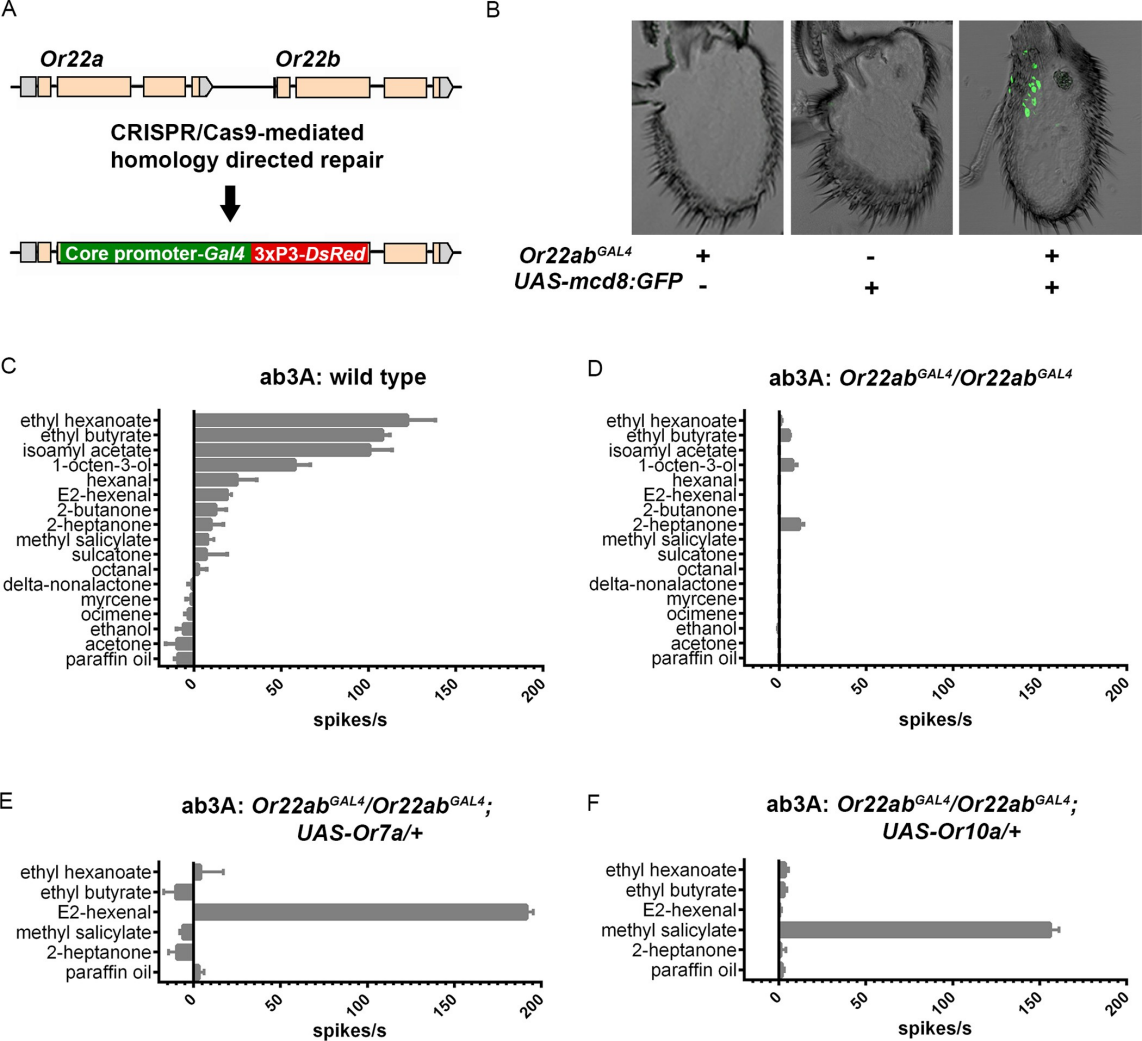
CRISPR/Cas9 knock-in of the *Gal4* gene into the *Or22a* and *Or22b* coding regions. (A) Genomic region of *Or22a* and *Or22b* before and after it is targeted for CRISPR/Cas9-mediated homology-directed repair to knock-in the *Gal4* transcription factor and DsRed eye marker genes. The formal designation of this stock is *Df(2L)Or22ab*, *TI{GAL4}Or22ab* but for convenience it is indicated as *Or22ab*^*GAL4*^. (B) GFP signal from antennal cross sections from *D*. *melanogaster* carrying the *Gal4* knock-in *(Or22ab*^*GAL4*^*)*, the *UAS-mcd8*:*GFP* transgene on the second chromosome, or both. (C, D) Odorant response profiles from (C) wild type control ab3A and (D) homozygous *Or22ab*^*GAL4*^ ab3A ORNs. (E, F) Odorant response profiles from *D*. *melanogaster UAS-Or* transgenes previously shown [15] to confer strong responses to E2-hexenal (Or7a) (E) or methyl salicylate (Or10a) (F), expressed in homozygous *Or22ab*^*GAL4*^ ab3A olfactory receptor neurons. In panels C-F neuronal responses are reported in spikes/second +/- S.E.M., using individual odorants diluted at 10^−2^ and presented for 0.5 seconds. n≥5 for all odorants tested. Spontaneous firing frequencies have been subtracted from all responses; responses to the paraffin oil diluent have been subtracted from the responses to all odorants.

We have now simplified this system by inserting a *GAL4* gene, as well as a *DsRed* eye marker, directly into the *Or22a*/*b* locus via CRISPR/Cas9 (Fig 7A, S2 Fig). This insertion can be readily maintained in homozygous form, without suffering the reduced viability of the previous deletion stock. Moreover, to drive expression in the empty neuron, this insertion chromosome needs to be combined with only one other construct, a *UAS-OrX*, rather than two, which greatly facilitates analysis.

To test this new version of the empty neuron system, we first crossed the *GAL4* insertion stock to a *UAS-GFP* stock and examined GFP expression. We detected labeling in the region of the antenna containing ab3 sensilla, as expected (Fig 7B). We then asked whether the insertion into the *Or22a/b* locus ablates the odor response of the ab3A neuron. Whereas in a control stock the ab3A neuron responded to a variety of odorants, including strong responses (>100 spikes/s) to ethyl hexanoate, ethyl butyrate and isoamyl acetate (Fig 7C), the insertion mutant showed little if any response to any tested odorant (Fig 7D).

We then expressed a *Drosophila UAS-Or7a* construct in the empty neurons. In the original empty neuron system, expression of Or7a conferred a very strong response to E2-hexenal but little if any response to ethyl hexanoate, ethyl butyrate, methyl salicylate, or 2-heptanone [15]. We found that in the new empty neuron system, Or7a likewise conferred a very strong response to E2-hexenal, but not the other odorants, (Fig 7E).

As a second test, we expressed the *Drosophila* Or10a receptor in the new empty neuron system and found a very strong response to methyl salicylate but not the other four odorants (Fig 7F), consistent with results obtained in the original system [15].

### An odor receptor for tsetse attractants

We then expressed a tsetse *Or*, *GmmOr35*, in the new empty neuron system, and tested it with a diverse panel of 26 odorants. The panel included compounds that emanate from tsetse host species and attract tsetse (*e*.*g*. acetone, 2-butanone, and 1-octen-3-ol), a tsetse repellent (delta-nonalactone), and a number of representative alcohols, ketones, aldehydes, esters, and terpenes that have been tested against odor receptors of other insects. Odorants were initially screened as 10^−2^ dilutions in paraffin oil, as in other studies.

**Fig 8:**
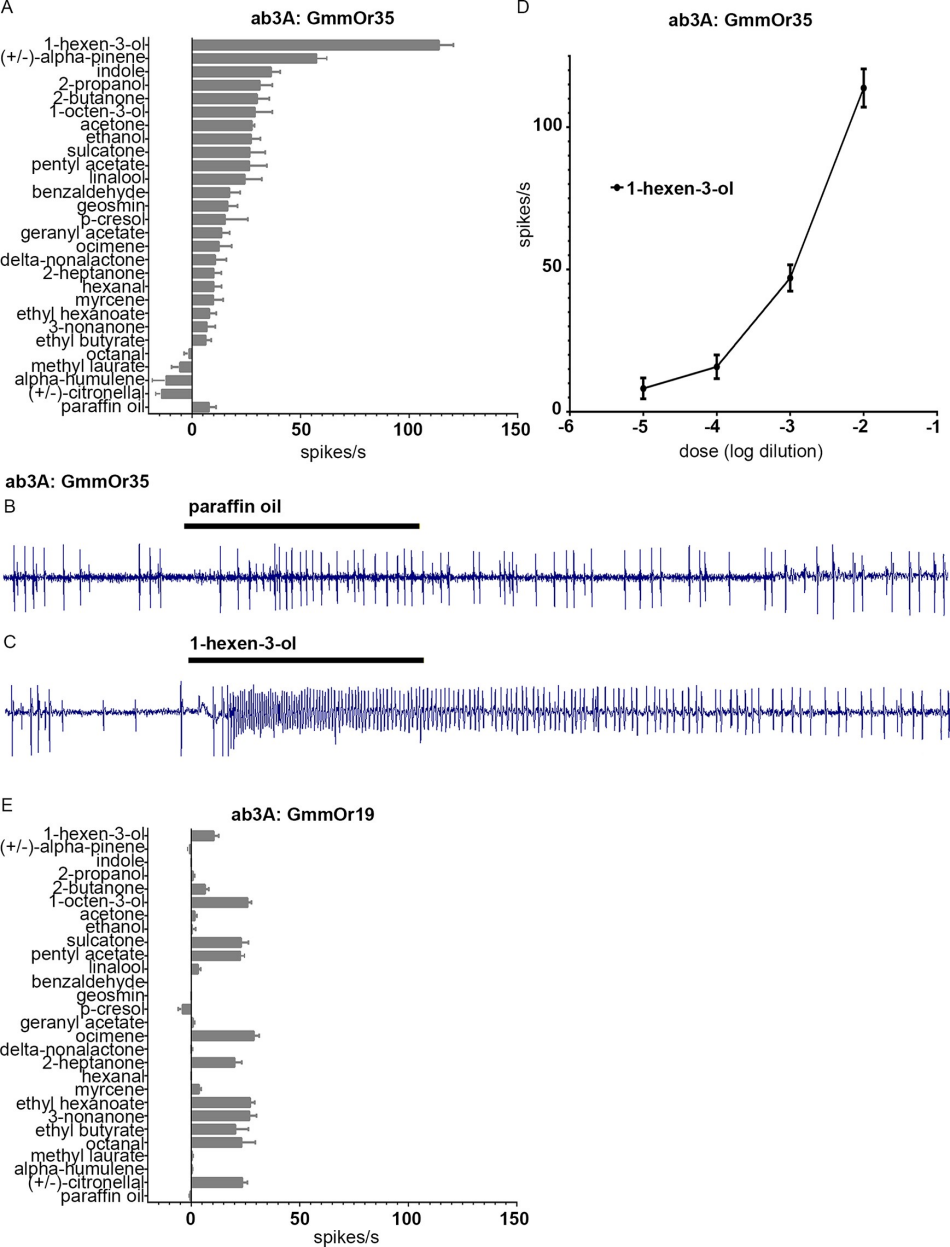
GmmOr35 responds strongly to 1-hexen-3-ol and moderately to alpha-pinene. (A) Odorant response profile of GmmOr35 expressed in homozygous *Or22ab*^*GAL4*^ ab3A ORNs. Neuronal responses are reported in spikes/second +/- S.E.M., to individual odorants diluted 10^−2^. (B,C) Sample traces from ab3A empty neurons expressing the *GmmOr35* transgene in response to the paraffin oil diluent (B) and 1-hexen-3-ol (C), each presented for 0.5 seconds (black bars). (D) Dose response curve for GmmOr35 and 1-hexen-3-ol. (E) Response profile of GmmOr19. In panels A, D, E, spontaneous firing frequencies have been subtracted from all responses; responses to the paraffin oil diluent have been subtracted from the responses to all odorants. n≥5 for all stimuli tested.

Expression of *GmmOr35* conferred a strong response to 1-hexen-3-ol, and more moderate responses to a variety of other odors (Fig 8A–8C). 1-hexen-3-ol is a human emanation and has previously been found to elicit a behavioral response from *G*. *morsitans* [34]. We confirmed the physiological response to 1-hexen-3-ol by testing a series of concentrations, and found that responses increased monotonically as a function of dose (Fig 8D). GmmOr35 also responds moderately (~60 spikes/s) to alpha-pinene, which elicited responses from other tsetse species in a coupled gas chromatography-electroantennogram study [35]. We note that there is evidence that alpha-pinene is produced by malaria parasites and is attractive to the *Anopheles* mosquitoes that transmit them [36].

Expression of another Or, GmmOr19, showed a different profile (Fig 8E). GmmOr19 conferred little if any response to 1-hexen-3-ol. Strong responses were not observed with any tested compound, but a variety of weak responses were elicited from GmmOr19. These results confirm that the responses conferred in this system are receptor-specific.

**Fig 9:**
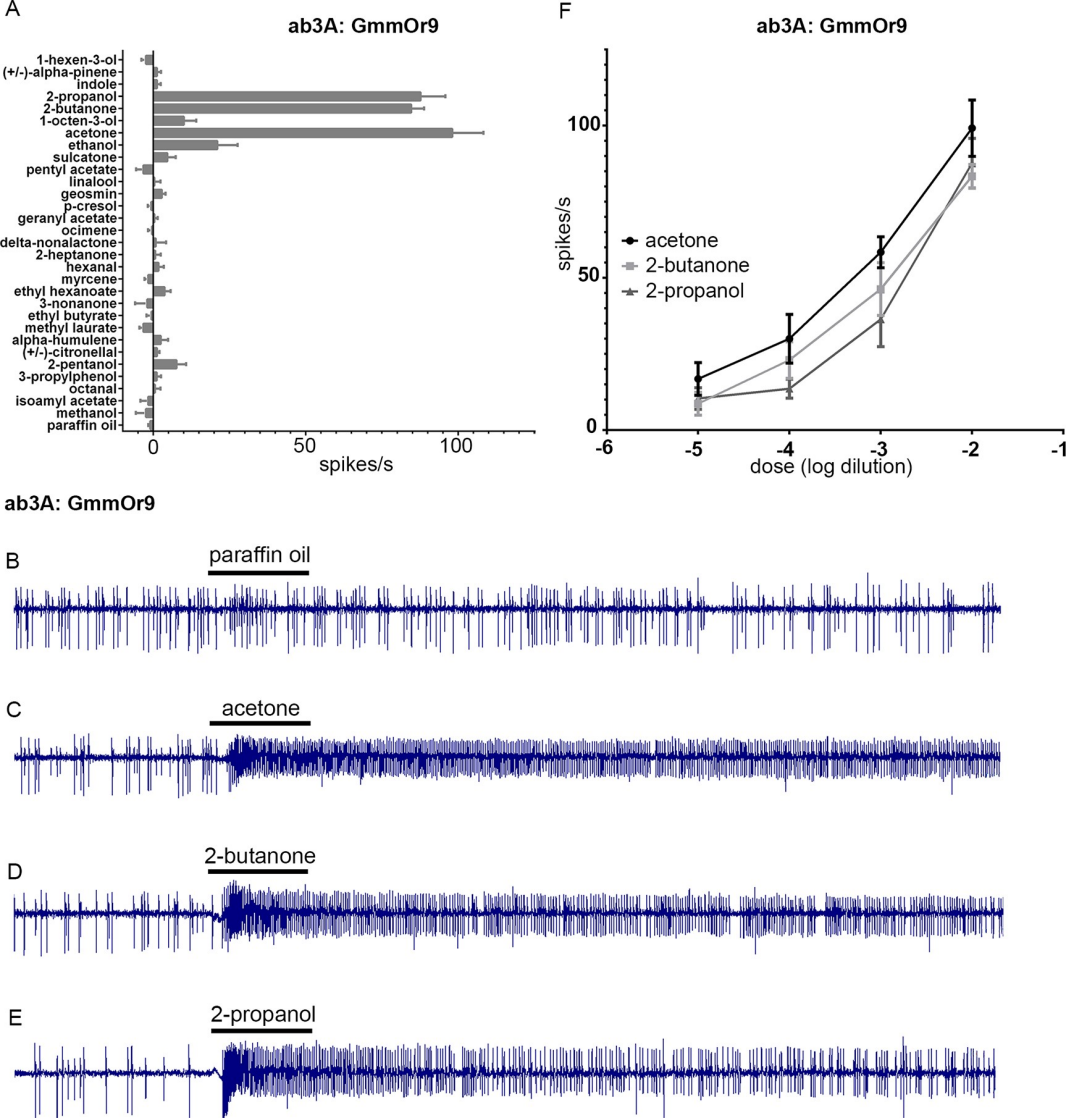
GmmOr9 responds strongly to 2-propanol, 2-butanone, and acetone. (A) Odorant response profile of GmmOr9 expressed in homozygous *Or22ab*^*GAL4*^ ab3A. (B-E) Sample traces. Black bars represent 0.5 second stimuli. (F) Dose response curves for GmmOr9. Error bars indicate +/- S.E.M. n≥5 for all stimuli tested.

We then tested GmmOr9, and found responses of ~100 spikes/s to three structurally related odorants: acetone, 2-butanone, and 2-propanol (Fig 9A–9E). We confirmed and extended these results via dose-response analysis for all three of these odorants (Fig 9F). Since acetone and 2-butanone are commonly used attractants in tsetse control, we were motivated to explore the cellular basis of this response in more detail.

**Fig 10:**
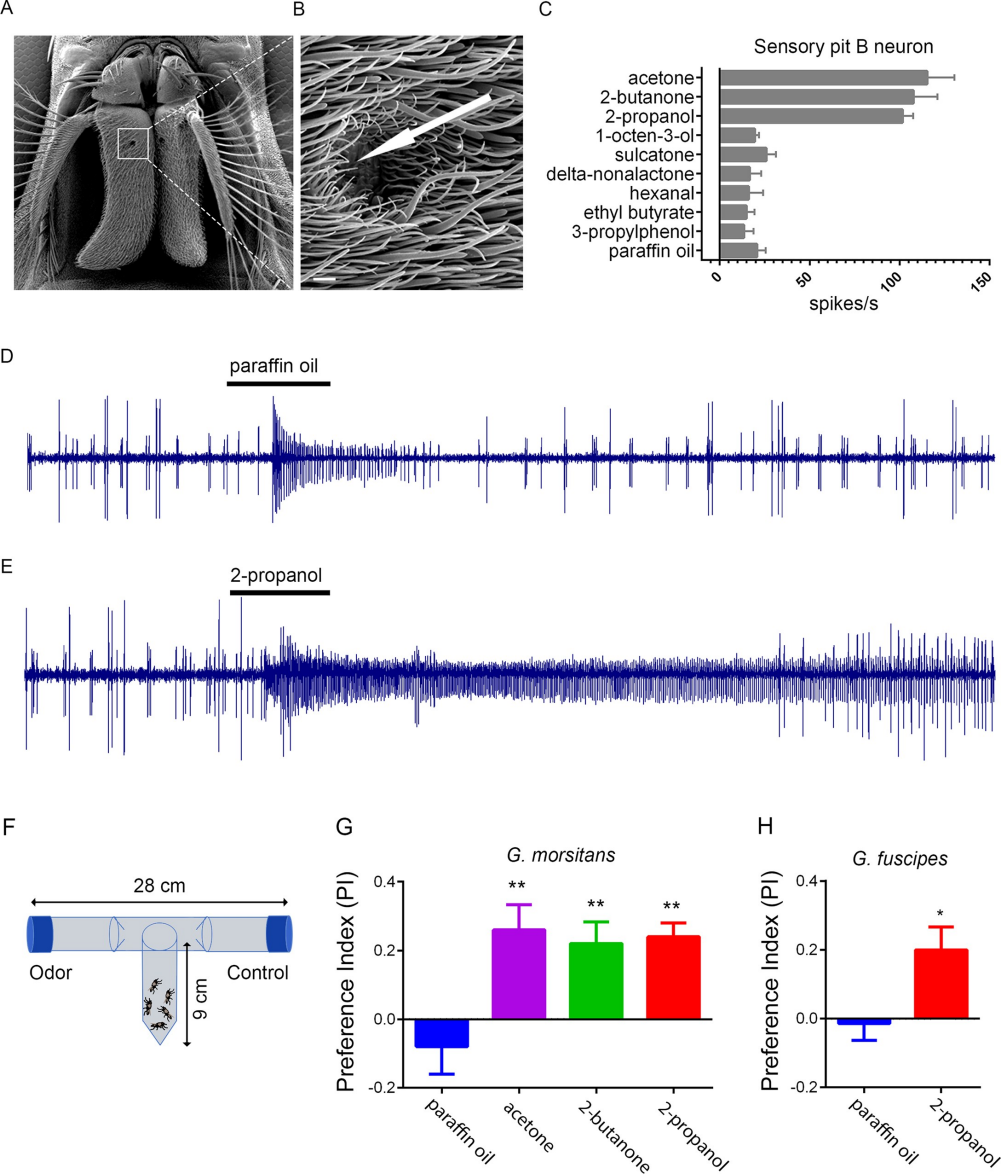
Responses of tsetse to acetone, 2-butanone, and 2-propanol. A) Scanning electron micrograph of the *G*. *morsitans* antenna. The white square is centered on the opening of the sensory pit. B) Magnified image of the opening to the sensory pit. The arrow indicates the approximate position of a recording electrode, which is inserted through the opening of the sensory pit in order to pierce a basiconic Type II sensillum for electrophysiological recording. Scale bar is 5 μm. (C) Odorant response profile of sensory pit B neurons. Odorants were diluted 10^−2^ in paraffin oil. n≥5 for all stimuli tested. (D,E) Sample traces from sensory pit sensilla in response to paraffin oil diluent (D) and 2-propanol (E) presented for 0.5 seconds (black bars). The B neuron, represented by the smaller spikes, shows a weak response to paraffin oil, a response that is also observed in some *Drosophila* neurons [46], and a strong response to 2-propanol. The A neuron, represented by large spikes, may be inhibited by 2-propanol. (F) The T-maze paradigm. (G) Behavioral responses of *G*. *morsitans*. Means were compared using one-way ANOVA, followed by Tukey’s test for pairwise comparison against paraffin oil for all odorants. **, p<0.01. n = 10 replicates for all odorants; n = 5 for the paraffin oil diluent. (H) Responses of *G*. *fuscipes*. t-test; p<0.05. n = 14 replicates.

*GmmOr9* is expressed in sensilla of the sensory pit (Fig 5B). Recording from the sensory pit is extremely challenging because these sensilla are not visible from outside the sensory pit. Nonetheless we made an effort to record from them by passing the recording electrode into the opening of the sensory pit and attempting to pierce sensilla without visual aid. The success rate was very low, and the recordings were not stable enough to record from a large number of odorants, but in >5 cases we were able to record physiological responses from acetone, 2-butanone and 2-propanol with sufficiently high signal-to-noise ratios that we could count spikes (Fig 10C–10E). In each case, we observed two distinct spike amplitudes, as if the recordings were from sensilla with two ORNs. In all cases, the ORN that produced the smaller spike, designated the B neuron, responded strongly to acetone, 2-butanone, and 2-propanol. Further analysis, and perhaps an alternative method of analysis, will be required to characterize these neurons in detail, but the strong responses to acetone and 2-butanone suggest that the sensory pit may play a role in the attractive response of tsetse to its animal hosts.

Is 2-propanol, like acetone and 2-butanone, attractive to tsetse? We tested *G*. *morsitans* in a T-maze and confirmed the results of field experiments that acetone and 2-butanone are attractive (Fig 10F and 10G). We also tested 2-propanol and found that it attracted *G*. *morsitans* as well, yielding a Preference Index (PI) very similar to those of acetone and 2-butanone when tested at the same 10^−3^ dilution. Finally, we asked whether 2-propanol would also attract a related species, *G*. *fuscipes*, which may account for the greatest number of cases of human African trypanosomiasis [37,38]. We found that 2-propanol attracted *G*. *fuscipes*, yielding a PI comparable to that of *G*. *morsitans* (Fig 10H).

## Discussion

We have examined the anatomical and molecular basis of olfactory function in the tsetse fly *G*. *morsitans*, which transmits debilitating disease to humans and animals in sub-Saharan Africa. The results are of interest both because of the health and economic impact of this disease vector, and also because of its unusual biology. We were particularly interested in comparing the anatomy and molecular organization of the tsetse antenna to that of the model organism *D*. *melanogaster*, which has been examined in great detail.

The *G*. *morsitans* antenna is like that of *D*. *melanogaster* in that it contains basiconic, trichoid, coeloconic, and intermediate sensilla (Figs 1 and 2). However, it is anatomically distinct in a variety of ways: i) its overall morphology is much more elongated; ii) it contains an invagination, the sensory pit, that is lacking in *D*. *melanogaster;* iii) its sacculus contains two chambers rather than three; iv) its basiconic sensilla are morphologically distinct from those of *D*. *melanogaster*, most notably in containing Type II and Type III sensilla in the sensory pit and sacculus, respectively. These two sensillum types have no counterparts in *D*. *melanogaster*.

We explored whether the morphological similarities and dissimilarities between the two species were accompanied by molecular likenesses and differences. First, we considered Obps, which have been characterized extensively in *D*. *melanogaster*. We tested tsetse orthologs of four *Obps* that are expressed in basiconic, trichoid, grooved coeloconic, or ungrooved coeloconic sensilla, respectively, in the *Drosophila* antenna. Each tsetse ortholog was expressed in the same morphological class of sensillum in *G*. *morsitans* (Fig 3). One interpretation of these results is that the developmental regulatory hierarchy that controls the most basic morphology of these sensilla also controls the expression of the *Obp* genes that they express.

We then investigated expression patterns of *Ors* in tsetse. As in *Drosophila*, individual *GmmOrs* are expressed in subsets of ORNs, with different *GmmOrs* showing different expression patterns. Of particular interest, *GmmOr9* and *GmmOr6* are expressed in neurons that innervate the sensory pit, a structure that does not exist in *Drosophila*.

Little is known about the function of sensory pits. The mapping of *GmmOr9* and *GmmOr6* to the tsetse sensory pit, together with direct electrophysiological recordings from sensilla of this pit (Fig 10), support an olfactory function for this structure. Sensory pits are found in antennae of other calyptrate flies besides tsetse, including the blow fly *Lucilia cuprina* and the flesh fly *Parasarcophaga dux* [39]. It will be interesting to determine whether sensory pits act in tracking food odors in these various fly species. In the hawkmoth, *Manduca sexta*, the third segment of the labial palp, a mouthpart appendage, contains a specialized pit called the labial pit organ. This organ is lined with sensilla that respond to carbon dioxide and have been hypothesized to be involved in the location of food sources or oviposition sites [40,41].

We simplified the empty neuron system (Fig 7), which should facilitate its use in analyzing other odor receptors from other organisms. This new system requires the combination of only two components, rather than three, and the viability of the new *Or* deletion is dramatically higher than the previous *Or* deletion. Taken together the new system is much more convenient to use than the original system.

Here, we used the new empty neuron system to examine the odor response profiles of GmmOr35, GmmOr19, and GmmOr9 (Figs 8 and 9). These three receptors all showed robust expression in subsets of ORNs located in various regions of the antenna. The three had different odor response profiles, of which one, GmmOr9, revealed a strong response to acetone, 2-butanone, and 2-propanol.

Acetone and 2-butanone emanate from bovine hosts of *G*. *morsitans* and are effective attractants in field studies. Artificial baits generally include either one or the other, but not both [6]; the two compounds are believed to fully substitute for each other [5,7]. Our work provides a potential explanation for such results. Perhaps the two odorants can substitute for each other because they both act via the same receptor, GmmOr9. This receptor might activate a circuit in tsetse that makes a major contribution to host-seeking behavior. At the same time, we note that other GmmOrs or other chemoreceptors may also respond to acetone or 2-butanone and contribute to host-seeking. In *G*. *palpalis* and *G*. *fuscipes*, Ouedraogo and den Otter [42] recently identified sensilla on the antennal surface that respond to acetone, suggesting that *G*. *morsitans* also could have additional sensilla and receptors outside the sensory pit that respond to acetone.

We found that 2-propanol also activates GmmOr9, and that it also attracts *G*. *morsitans*. Moreover, 2-propanol attracts *G*. *fuscipes*, which is likely responsible for the greatest number of cases of African sleeping sickness [37,38]. These results identify 2-propanol as a potential attractant for field use. This compound, also known as isopropanol, or rubbing alcohol, has many virtues as a field agent: it is relatively inexpensive, environmentally friendly, and non-toxic.

Taken together, our results illustrate how molecular and genetic analysis of the tsetse olfactory system may be useful in informing field studies of this organism. The results suggest the possibility of screening a wide variety of compounds to identify other odorants that activate GmmOr9 strongly, and then testing them in the field as baits for traps or targets. Such an approach could be useful in controlling an insect disease vector that has imposed a great burden on sub-Saharan Africa for centuries.

## Materials and methods

### Tsetse flies

*G*. *m*. *morsitans* were maintained in the Yale insectary at 24°C with 50–55% relative humidity. All flies received defibrinated bovine blood every 48 hours using an artificial membrane feeding system [43].

### Scanning electron microscopy

Whole fly heads were fixed in a solution of 0.1 M sodium cacodylate, 2% paraformaldehyde and 2.5% glutaraldehyde for 2 hrs in microporous specimen capsules (Electron Microscopy Sciences). Heads were then dehydrated in a graded series of ethanol washes until they were incubated overnight in 100% ethanol. Ethanol-dehydrated heads were dried in a Leica CPD300 critical point dryer. Antennae were removed from dried heads with forceps and then glued to metallic pegs with graphite conductive adhesive (Electron Microscopy Sciences). Samples were then coated in 2 nm of iridium with a Cressington Sputter Coater and imaged in a Hitachi SU-70 SEM. For sample preparation of antennal cross sections of the sensory pit and sacculus, 10 μm cross sections were adhered directly to metallic pegs and immediately coated in iridium for imaging.

### Identification of *D*. *melanogaster* and *G*. *morsitans Obp* ortholog pairs

*D*. *melanogaster* Obp protein sequences were taken from FlyBase and were individually queried against the *G*. *morsitans* peptide library on VectorBase using BLASTp. The top GmmObp results were queried back against the *D*. *melanogaster* genome using DELTA-BLAST. In each case the reciprocal BLAST search identified the same *D*. *melanogaster* Obp that was originally queried, confirming the ortholog pairing. The percent sequence identity ranged from 39–53% and the E-values from DELTA-BLAST ranged from 2e^-39^ to 2e^-26^ for the ortholog pairs.

### RNA FISH and immunohistochemistry

RNA was extracted from dissected antennae with RNeasy Mini Kit (Qiagen) and converted into antennal cDNA by the Superscript iii Reverse Transcriptase kit (Thermo Fisher) according to the manufacturer’s instructions. cDNA from genes of interest was then amplified by PCR (see S1 Table for primer sequences) and cloned into the pGEM-T Easy Vector System (Promega) for digoxigenin (DIG)-labeled RNA antisense probe synthesis using standard methods. The protocol for FISH is similar to that described in [24], except for the following modifications: male and female antennae were cryosectioned at either 40 or 10 μm, RNA probes were hybridized at 55°C overnight, sheep anti-DIG-POD primary antibodies were incubated for 45 minutes for Tyramide Signal Amplification (TSA) with Cy3 (PerkinElmer). When mouse anti-ELAV immunohistochemistry was used in conjunction with TSA, after the TSA reaction was completed slides were washed with PBST for 2x10 min. and incubated in a 5% Western blocking solution (Roche) in PBST for 30 min. The stock mouse anti-ELAV was diluted in 5% blocking solution (1:50) and added to slides for overnight incubation at ~4°C. Slides were washed in PBST 3x5 min. and anti-ELAV was detected with goat anti-mouse 488 (Thermo Fisher) diluted 1:500. The anti-ELAV (ELAV-9F8A9) antibody, developed by G. Rubin, was obtained from the Developmental Studies Hybridoma Bank, created by the NICHD of the NIH and maintained at The University of Iowa, Department of Biology, Iowa City, IA 52242. Microscopy was performed using either a Carl Zeiss LSM 880 or 510 Laser Scanning Confocal Microscope and images were processed with ImageJ software.

### *Or22a*^*-*^*b*^*-*^ *Gal4* line

For the expression of gRNAs, the genomic sequence flanking the 5’ (second exon of *Or22a*) and 3’ (second exon of *Or22b*) cut sites were cloned into the pCFD4 plasmid, a gift from Simon Bullock (Addgene # 49411). gRNAs were cloned into pCFD4 directly by PCR using Q5 polymerase (New England Biolabs), pCFD4 as a genomic template, and the following primers that contain the desired gRNA sequences: 5’TATATAGGAAAGATATCCGGGTGAACTTCGAGATTGGAGTCAACATGTAGTTTTAGAGCTAGAAATAGCAAG3’ and 5’ATTTTAACTTGCTATTTCTAGCTCTAAAACACTGCGTTCGAGATCACCGCGACGTTAAATTGAAAATAGGTC3’. The PCR product, which contains both gRNA templates, was run on a gel and the resulting 600 bp band was gel-purified. In parallel, the original pCFD4 plasmid was digested with BbsI and the linearized plasmid was also gel-purified. The gel-purified products were annealed by Gibson Assembly (NEB) according to the manufacturer’s instructions. The donor vector used for homology-directed repair was pHD-DsRed-attP, a gift from Melissa Harrison & Kate O’Connor-Giles and Jill Wildonger (Addgene #51019), with the *Drosophila* synthetic core promoter and Gal4 sequence from the pBPGUW plasmid, a gift from Gerald Rubin (Addgene #17575), cloned into 5’ MCS. 1 kb length homology arms were PCR-amplified from Canton-S gDNA using the following primers: homology arm 1 primers 5’GGCCTTTCGCCAGGACACTCGATGCACG3’ and 5’TGTACAAGAAAGCTGAACGAATGTTGACTCCAATCTCGAG3’; homology arm 2 primers 5’TTCCGTCAATCGAGTTCAAGGCGTTCGAGATCACCGCTTG3’ and 5’TTGTGTCGCCAACCCGCATCCGCATCCT3’. The homology arms were cloned into the modified pHD-DsRed-attP donor vector by Gibson Assembly. The finalized gRNA expression plasmid and donor vector were injected into *yw;nos-Cas9(III-attP2)* embryos by BestGene Inc. (Chino, CA).

### *UAS* lines

*GmmOrs* were cloned into the pJFRC81-10XUAS-IVS-Syn21-GFP-p10 (pJFRC81) vector, which was a gift from Gerald Rubin (Addgene plasmid # 36432). *GmmOrs* were first amplified from antennal cDNA by PCR. 5’ and 3’ *GmmOr* PCR primers were designed with 20bp overhangs that overlapped with flanking regions of the GFP coding region within pJFRC81. The pJFRC81 vector was linearized by PCR using primers that flanked GFP and amplified outwards thereby removing the coding region of GFP. Using Gibson assembly (NEB) the linearized vector lacking GFP and the cloned *GmmOr* were assembled into a plasmid for bacterial transformation. Modified pJFRC81 vectors were injected into either *y*^*1*^ *w67c23; P{CaryP}attP2* or *M{3xP3-RFP*.*attP}ZH-86Fb* embryos by BestGene Inc. for PhiC31 integration.

### Electrophysiology

Extracellular single-sensillum recordings (SSRs) were performed essentially as described in [44]. *D*. *melanogaster* flies were housed in incubators set to 25°C and 50% humidity prior to recording, and only fruit flies aged 3–5 days old were used for SSR. For SSR in *G*. *morsitans* a similar approach was taken except for the following: male and female flies were aged 5–10 days, flies were immobilized in 1000 vs. 200 μl pipette tips, and the ground electrode was placed in the eye or pierced though the cuticle at the end of the antenna. *G*. *morsitans* were maintained in Yale’s insectary at 24°C with 50–55% relative humidity. All tsetse flies received defibrinated bovine blood (Hemostat Laboratories) every 48 hours through an artificial membrane feeding system [43]. Chemicals used for odorant delivery were purchased from Sigma-Aldrich and were of a purity ≥98%. Chemicals were dissolved at dilutions of 10^−5^ to 10^−2^ in paraffin oil. To prepare odor cartridges, 25 μl of diluted chemical was applied to a 13 mm diameter filter disc paper inside a Pasteur pipette that was then capped with a 1000 μl pipette tip and allowed to equilibrate for 10 min prior to use. Odor cartridges were used for no more than two presentations. Odorant stimuli were presented by placing the tip of the cartridge into a glass tube delivering a stream of humidified air (~2000 ml/min) to the fly antenna, and administering a 500 ms pulse of air (~200 ml/min) through the cartridge. The neuronal firing rate was measured 100 ms after the onset of the odor pulse to compensate for the time it takes for the odor to reach the antenna from the odor cartridge. To calculate the neuronal firing rate for a given odorant, the rate measured from paraffin oil presentation alone and the baseline firing rate obtained pre-odorant delivery were subtracted from the rate obtained after odorant delivery. Neuronal firing rates were recorded and measured in AUTOSPIKE (Syntech) and plotted in Prism (GraphPad). ANOVA statistical analysis were performed using PAST software (version 2.17c; http://folk.uio.no/ohammer/past/).

### T-maze paradigm

We adapted a previously published T-maze paradigm [18]. The T-maze was constructed from three disposable 50 ml screwcap polypropylene tubes and an 11 cm length of PVC tubing of internal diameter 2.5 cm (Fig 10D). Two of the tubes contained holes, 1 cm in diameter, at their distal tips. A filter disc containing 100 μl of odorant was placed on the inner surface of the plastic cap of one of tube; 100 μl of the paraffin oil diluent was placed likewise on the cap of the other tube. A third 50 ml tube containing 5 male flies was fitted through a hole in the PVC tube. After 60 min the number of flies (O) in the 50 ml tube containing the odor and the number (C) in the control tube were scored. A Preference Index (PI) was calculated following an established convention [45] as (O-C)/T, where T is the total number of flies used in the trial, *i*.*e*. 5. A score of 1 represents complete attraction. Testing was at 23°C and 50% relative humidity. The flies were 8d old, had been fed twice, and were tested 72h after the last feeding.

## Supporting information

S1 Fig Fluorescent *in situ* hybridization of anti-sense and sense negative control RNA probes to cross sections of the *G. morsitans* antenna. All confocal images were taken with similar laser power and digital gain settings. (A, B) *GmmObp59a*, (C, D) *GmmOrco*, (E, F) *GmmOr9*, (G, H) *GmmOr35*, (I, J) *GmmOr19*, (K, L) *GmmOr44*.
(TIF)

S2 Fig Validation of CRISPR/Cas9 knock-in of the *Gal4* transcription factor gene into the *Or22a* and *Or22b* coding regions. (A) Genomic region before and after it is targeted for CRISPR/Cas9-mediated homology-directed repair to knock in the *Gal4* transcription factor and DsRed eye marker genes. The relative locations of PCR primers used for validating the insertion of the construct are shown below along with the expected product sizes. (B) PCR amplification products from primer sets 1 and 2 using genomic DNA from wild type or homozygous *Or22ab*^*GAL4*^ flies. (C) Sequence of second exon of *Or22a* from a wild type genome versus the sequence from PCR-amplified DNA using primer set 1 and genomic DNA from homozygous *Or22ab*^*GAL4*^ flies. Base pairs in green indicate the sequence of the 5’ portion of the transgene.
(TIF)

S1 Table PCR primers used to amplify *GmmOrs* and *GmmObps* from cDNA. (XLSX)
