# Supplementary figures and images for: The molecular and cellular basis of olfactory response to tsetse fly attractants
Source: PLoS Genet. 2019 Mar 15;15(3):e1008005. doi: 10.1371/journal.pgen.1008005 (PMC6420007; doi:10.1371/journal.pgen.1008005)

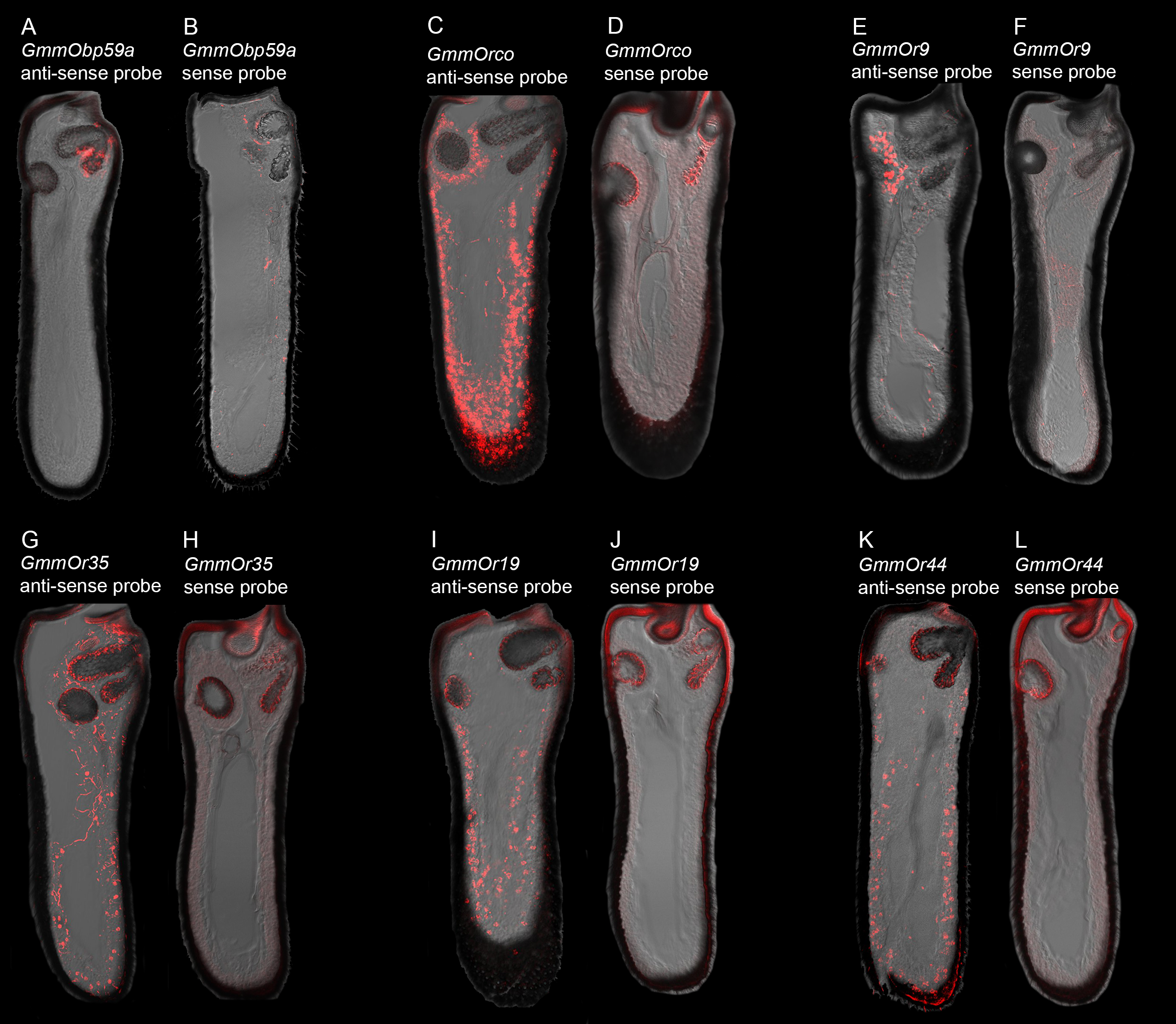

Supplement: S1 Fig — All confocal images were taken with similar laser power and digital gain settings. (A, B) GmmObp59a, (C, D) GmmOrco, (E, F) GmmOr9, (G, H) GmmOr35, (I, J) GmmOr19, (K, L) GmmOr44. (TIF) [file pgen.1008005.s001.tif]

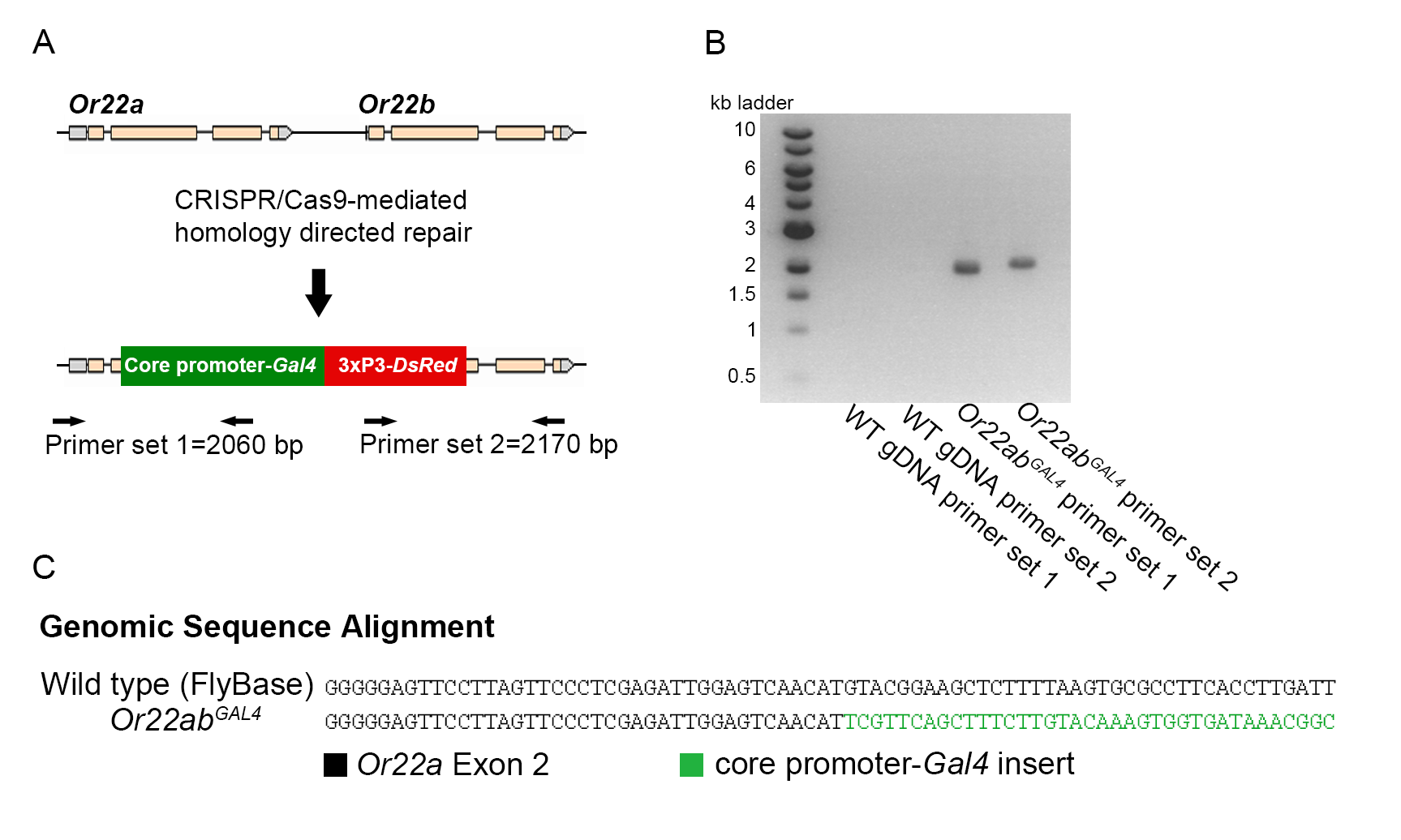

Supplement: S2 Fig — (A) Genomic region before and after it is targeted for CRISPR/Cas9-mediated homology-directed repair to knock in the Gal4 transcription factor and DsRed eye marker genes. The relative locations of PCR primers used for validating the insertion of the construct are shown below along with the expected product sizes. (B) PCR amplification products from primer sets 1 and 2 using genomic DNA from wild type or homozygous Or22abGAL4 flies. (C) Sequence of second exon of Or22a from a wild type genome versus the sequence from PCR-amplified DNA using primer set 1 and genomic DNA from homozygous Or22abGAL4 flies. Base pairs in green indicate the sequence of the 5’ portion of the transgene. (TIF) [file pgen.1008005.s002.tif]
